# Association News

**Published:** 1897-06

**Authors:** 


### ASSOCIATION NEWS.

The Ontario Veterinary Association, through a delegated representative party of the State Association, have asked to have the following inserted in their Act of Incorporation: "It shall not be lawful for any person not registered to practise veterinary medicine or surgery, or to perform any surgical operation whatever for hire, gain, or hope of reward. And, if any person not registered pursuant to this Act, for hire, gain, or hope of reward, practises or professes to practise veterinary medicine or surgery or advertises to give advice in veterinary medicine or surgery, he shall, upon summary conviction thereof, before any Justice of the Peace, for each and every offence, pay a penalty not exceeding $25 nor less than $5."

The Perth Veterinary Medical Association of Ontario recently held a meeting and elected the following officers: President, Dr. Gibb, of St. Mary's; Vice-President, Dr. Hodgins, Stratford; Secretary, Dr. Wagner; Treasurer, Dr. Tavistock. Some papers were read and discussed, and Drs. William Gibb, Steele, Clark, G. Gibb, Orr, and Shillington were authorized to represent this Association as delegates at the Ontario Veterinary Association held at Toronto on March 25th. Dr. Hutchings, of Mitchell, described a case of choke in a cow, the result of an old shoe lodging in the anterior part of the œsophagus. Similar cases were reported by Drs. Hodgins and Gibbs. An interesting paper was then read by Dr. Gibbs on lameness in all forms affecting the different extremities, giving a concise line of differential symptoms, and referred to fractures and modes of bandaging the same.

The Schuylkill Valley Veterinary Medical Association will meet at the Hotel Woll, Pottsville, Wednesday, June 16, 1897.

